# Inapparent maternal ZIKV infection impacts fetal brain development and postnatal behavior

**DOI:** 10.1371/journal.ppat.1013850

**Published:** 2026-01-12

**Authors:** Tsui-Wen Chou, Micheal McCourt, Eduard Marmut, Vishal Karuppusamy, Marissa Lindman, Irving Estevez, Benjamin D. Buckley, Joshua K. Thackray, Kailyn Rodriguez, Marialaina Nissenbaum, Charul Mishra, Colm Atkins, Alexander W. Kusnecov, Max A. Tischfield, Brian P. Daniels

**Affiliations:** 1 Department of Cell Biology and Neuroscience, Rutgers University, Piscataway, New Jersey, United States of America; 2 Department of Genetics, Rutgers University, Piscataway, New Jersey, United States of America; 3 The Human Genetics Institute of New Jersey, Rutgers University, Piscataway, New Jersey, United States of America; 4 W.M. Keck Center for Collaborative Neuroscience, Rutgers University, Piscataway, New Jersey, United States of America; 5 Department of Psychology, Rutgers University, Piscataway, New Jersey, United States of America; Washington State University, UNITED STATES OF AMERICA

## Abstract

Zika virus (ZIKV) has emerged as a significant public health concern due to its association with severe neurological outcomes in infants, including microcephaly and congenital Zika syndrome (CZS). However, while the majority of ZIKV infections during pregnancy do not result in CZS, the potential long-term neurological effects of mild or inapparent maternal infections remain poorly understood. In this study, we adapted a model of maternal ZIKV infection in human *STAT2* knock-in (*hSTAT2*) mice to investigate the effects of ZIKV infection during mid-gestation, aiming to mirror typical asymptomatic infections as they occur in humans. We found that maternal ZIKV infection at mid-gestation leads to vertical transmission without causing overt developmental deficits or clinical signs in dams or offspring. Despite the absence of immediate clinical signs, transcriptomic analyses revealed significant changes in the developing fetal brain, particularly in genes related to synaptic function and neuronal development. These molecular alterations were associated with increased synaptic density in the hippocampus and heightened susceptibility to chemically induced seizures in offspring, suggesting subtle yet significant long-term neurological consequences. Using motion sequencing (MoSeq), an unsupervised machine learning approach that profiles naturalistic motor behavior, we also identified persistent, sex-biased alterations in the content and structure of spontaneous behavior in offspring exposed to maternal ZIKV infection. Our findings highlight that even mild maternal ZIKV infections can disrupt fetal neurodevelopment, underscoring the need for enhanced monitoring and public health measures for children exposed to ZIKV in utero but who do not experience severe developmental alterations at birth. Additionally, our study provides a valuable animal model and comprehensive, cell type-specific transcriptomic datasets that will facilitate new lines of investigation into the impact of inapparent maternal ZIKV infections on fetal and childhood brain development.

## Author summary

Zika virus infection during pregnancy is best known for causing severe brain and developmental abnormalities in a subset of infants. However, most exposed children are born without obvious problems, and the long-term consequences of these more common cases remain unclear. Here we used a mouse model that mimics mild, asymptomatic infection in the mother to ask whether such exposures can still alter brain development in offspring. We found that maternal infection allowed virus to reach the fetal brain without causing reduced brain size. Nevertheless, fetal brains showed broad changes in patterns of gene activity that guide how nerve cells grow and connect. As the offspring matured, they developed more excitatory synapses, became more susceptible to chemically induced seizures, and showed lasting, sex-specific changes in spontaneous behavior. Our findings suggest that even mild maternal Zika virus infections may carry hidden risks for later neurological or behavioral difficulties. These findings support the need for long-term monitoring of exposed children.

## Introduction

Zika virus (ZIKV), a mosquito-borne flavivirus, emerged as a significant public health concern during an outbreak in the Americas in late 2015 [1]. Over the past decade, ZIKV has gained global attention, primarily due to its association with severe neurological outcomes in infants, including microcephaly [2,3]. In addition to microcephaly, congenital Zika syndrome (CZS) encompasses a range of profound developmental and neurological abnormalities resulting from maternal-fetal transmission of ZIKV [4,5]. However, while severe cases of CZS have highlighted significant risks associated with ZIKV infection during pregnancy, epidemiological evidence indicates that the majority of ZIKV infections do not result in CZS. Instead, most infants exposed to ZIKV during gestation appear clinically normal at birth [6–9]. Despite representing the majority of human cases, however, the potential long-term neurological effects of mild or “inapparent” ZIKV infections during pregnancy are not well understood.

In adults, ZIKV infection is often asymptomatic or presents with mild, nonspecific symptoms such as fever, rash, and conjunctivitis [6,10]. Despite the lack of severe illness in most cases, recent evidence suggests that even mild systemic viral infections during pregnancy can have significant impacts on fetal development. For example, infections with cytomegalovirus, rubella virus, and influenza virus during pregnancy have been associated with a range of developmental deficits in offspring, including neurological impairments and increased risk of neuropsychiatric disorders [11–17]. Despite these insights, the effects of mild maternal ZIKV infection on neurodevelopment and long-term outcomes in offspring have not been adequately studied, leaving a critical gap in our understanding of the full spectrum of clinical outcomes associated with ZIKV infection during pregnancy.

To date, animal models have been essential for studying ZIKV pathogenesis and maternal-fetal transmission. Mouse models, in particular, have provided valuable insights but also present significant limitations. Wild-type adult mice are resistant to ZIKV infection, primarily due to species-specific differences in type I interferon (IFN) signaling arising from the inability of ZIKV to antagonize human, but not murine, STAT2 [18–20]. To overcome this limitation, past work has heavily utilized immunodeficient mouse lines lacking components of the type I IFN response, including the IFN receptor IFNAR1 and transcription factors such as interferon regulatory factor (IRF)3, IRF5, and IRF7 [18,21–24]. While these models are susceptible to ZIKV infection, they often exhibit severe disease phenotypes that do not reflect the typical mild or asymptomatic infections experienced in humans. To create a more representative model, Gorman et al. developed a human *STAT2* knock-in (*hSTAT2*) mouse line, in which murine *Stat2* is replaced with human *STAT2* [25]. This model allows ZIKV to antagonize STAT2-mediated innate immune signaling, rendering mice susceptible to subcutaneous ZIKV infection in an immunocompetent background.

In this study, we adapted the *hSTAT2* mouse model to investigate the effects of maternal ZIKV infection during mid-gestation, aiming to more closely model inapparent or mild maternal infections as they typically occur in humans. We infected pregnant *hSTAT2* dams at mid-gestational time points and demonstrate that maternal ZIKV infection during this period leads to vertical transmission without causing overt developmental deficits or clinical signs of disease in either dams or their offspring. Despite the absence of immediate clinical signs, we show that inapparent maternal infection induces transcriptomic changes in the developing fetal brain, particularly affecting genes related to synaptic function and neuronal development. These transcriptomic alterations are associated with phenotypic changes in the hippocampus, including increased synaptic density in the dentate gyrus. Furthermore, we demonstrate that offspring from infected dams exhibit increased susceptibility to chemically induced seizures and alterations to spontaneous motor activity, suggesting long-term functional consequences of inapparent maternal ZIKV infection. Our findings highlight the potential for subtle yet significant neurological impacts of mild maternal ZIKV infection, underscoring the need for further research on the developmental consequences of such cases, which represent the vast majority of ZIKV infections. Given the risk of long-term functional changes even in the absence of overt clinical symptoms, enhanced monitoring and other public health measures may be warranted to better track and support children following in utero ZIKV exposure.

## Results

### hSTAT2 mice are suitable for a model of inapparent maternal ZIKV infection

**Fig 1:**
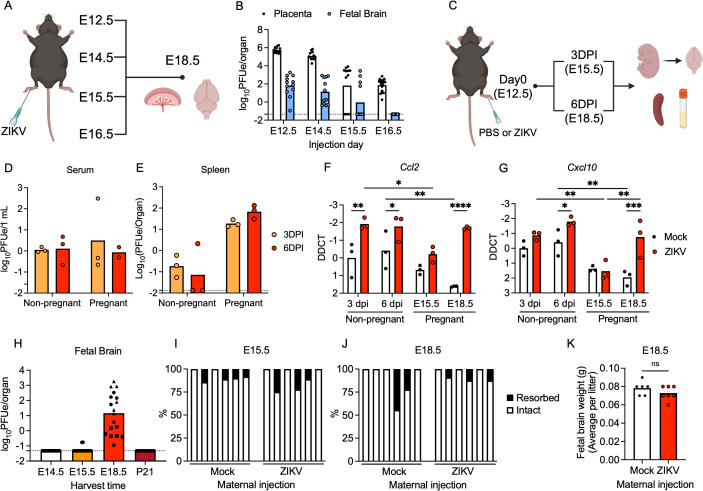
hSTAT2 mice are suitable for a model of inapparent maternal ZIKV infection. **(A)** Schematic of experimental design testing the impact of gestational stage on viral RNA abundance in placenta and fetal brains. **(B)** Abundance of viral RNA in placenta and fetal brains harvested at E18.5 was measured by RT-qPCR. Dashed line indicates the limit of detection. N = 12-18 mice per time point. **(C)** Schematic of experimental design testing viral RNA abundance and inflammatory gene expression in maternal and fetal tissues following maternal infection at E12.5. **(D-E)** Viral RNA was measured in maternal serum **(D)** and spleen **(E)** samples at 3 and 6 dpi via RT-qPCR. Dashed line indicates the limit of detection. N = 3 mice per condition. **(F-G)** Expression profiles of Ccl2 (F) and Cxcl10 **(G)** in spleens from pregnant dams and age matched, non-pregnant female littermates as assessed by RT-qPCR analysis on 3 and 6 dpi. N = 3 mice per condition. **(H)** Viral RNA abundance in fetal brains harvested on indicated embryonic/postnatal days was measured by RT-qPCR. Dashed line indicates the limit of detection. Shapes indicate distinct litters from which individual pups were derived within each condition. N = 8-18 mice per time point. **(I-K)** Fetal resorption rates observed on E15.5 **(I)** and E18.5 **(J)** and fetal brain weight on E18.5 **(K)** following maternal infection at E12.5. N = 5-7 mice per litter and 6-7 litters per group. * p < 0.05, ** p < 0.01, *** p < 0.001. Panels A and C were created in BioRender. Daniels, B. (2026). https://BioRender.com/pwulc2b and https://BioRender.com/x7sbpg9.

To assess the potential impact of asymptomatic or otherwise “inapparent” maternal infection with ZIKV, we adapted a model of maternal ZIKV infection in *hSTAT2* knock-in mice [25] in order to identify conditions under which ZIKV could reach fetal brains without causing overt developmental deficits. Based on previous studies showing a correlation between infection during early gestation and the severity of fetal ZIKV pathogenesis [10], we hypothesized that infecting dams during middle to late stages of gestation would allow us to capture an intermediate phenotype in which vertical transmission occurs but does not result in clinical signs associated with CZS. We thus infected pregnant *hSTAT2* dams at different embryonic days (E12.5, E14.5, E15.5, and E16.5) and harvested fetal brains and placentas at E18.5 (Fig 1A). ZIKV RNA was detectable in nearly all placentas at E18.5, irrespective of the timing of maternal infection (Fig 1B). In contrast, fetal brains exhibited an inverse relationship between gestation time at the point of maternal infection and the detection of ZIKV RNA in the fetal brain at E18.5. Of the timepoints we tested, we noted that maternal infection at E12.5 yielded the highest and most consistently detectable levels of viral RNA in the fetal brain. We thus selected E12.5 as the most suitable time point for further development of our model of inapparent maternal ZIKV infection.

To confirm that maternal infection at E12.5 resulted in vertical transmission without causing overt clinical signs of disease in either offspring or dams, we subcutaneously infected pregnant *hSTAT2* dams and age-matched, non-pregnant *hSTAT2* female littermate controls with ZIKV and collected maternal tissues at 3 days post-infection (dpi) (E15.5) and 6 dpi (E18.5) for analysis (Fig 1C). ZIKV RNA was detected in the serum and spleen of both pregnant and non-pregnant female mice at both time points, indicating successful and ongoing infection of maternal tissues (Fig 1D-1E). Consistent with the detection of active infection, we observed robust upregulation of inflammatory cytokines and chemokines in the spleens of infected dams (Figs 1F-1G and S1). Notably, pregnant dams exhibited an overall trend of lower cytokine and chemokine expression in splenic tissues compared to non-pregnant littermate controls, due perhaps to pregnancy-associated immunosuppression [26–29]. Together, these data confirm that subcutaneous infection at E12.5 in pregnant, immunocompetent *hSTAT2* mice results in limited but detectable systemic ZIKV infection and immune activation in maternal tissues, similar to previous studies that performed subcutaneous infections at earlier stages of gestation [21,25].

In addition to confirming successful infection of maternal tissues, we also profiled the kinetics of vertical transmission to fetal brain tissue, noting that viral RNA was not detectable in fetal brain within the first 72 hours after maternal infection at E12.5, but was consistently detectable by E18.5 in all fetal brains tested (Fig 1H). Infection of the fetal brain was apparently self-limiting, as ZIKV RNA could no longer be detected in the brains of post-natal day 21 (P21) weanlings born to dams infected at E12.5. To assess whether this transient CNS infection resulted in clinical signs of disease or developmental abnormality in offspring, we measured both rates of fetal resorption during pregnancy as well as the brain weights of offspring born to dams infected at E12.5. Infected dams exhibited rates of fetal resorption at E15.5 and E18.5 that were indistinguishable from mock-infected pregnant littermate controls (Fig 1I-1J). Similarly, brain weights of neonates born to infected dams did not differ from those born to control dams (Fig 1K). These data confirm that infection of *hSTAT2* damns during mid-gestation (E12.5) results in vertical transmission without inducing the gross deficits in fetal growth or survival observed in models using immunodeficient mouse lines or in those infecting pregnant dams at earlier stages of gestation [21,30,31]. We thus moved forward using this paradigm as a suitable system for studying inapparent maternal ZIKV infection, in which ZIKV crosses the placental barrier to infect the fetal brain without inducing apparent clinical disease in offspring.

### Inapparent maternal ZIKV infection impacts the transcriptome of fetal brain tissue

**Fig 2:**
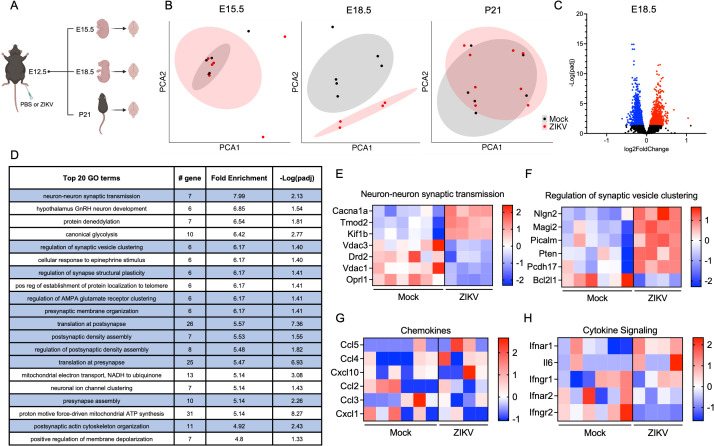
Inapparent maternal ZIKV infection dysregulates synaptic-related pathways in fetal brains. **(A)** Schematic of experimental design for bulk RNA-seq analysis of fetal brain tissue. **(B)** Principal component analysis (PCA) derived from bulk RNA-seq of offspring brains harvested at indicated time points following maternal infection on E12.5. N = 4-8 mice per condition per time point. **(C)** Volcano plot illustrating differential expression of transcripts in E18.5 fetal brains. **(D)** Top 20 enriched GO terms obtained from GO enrichment analysis of DEGs in E18.5 fetal brains. Synaptic-related GO terms are highlighted in blue. **(E-F)** Heatmaps depicting relative expression values (z scores) of genes associated with selected synaptic-related GO terms: neuron-neuron synaptic transmission **(E)** and regulation of synaptic vesicle clustering **(F)**. **(G-H)** Heatmaps depicting relative expression values of genes associated with chemokines **(G)** and cytokine signaling **(H)**. Panel A was created in BioRender. Daniels, B. (2026). https://biorender.com/s6nbah9.

To better understand the outcomes of fetal brain infection in our model, we investigated whether maternal ZIKV infection induced transcriptomic changes in fetal brains. Pregnant dams were infected with ZIKV at E12.5, and brains of offspring were harvested at E15.5, E18.5, and P21 for bulk RNA sequencing (RNA-seq) analysis (Fig 2A). Principal component analysis revealed distinct clustering of fetal brain samples derived from infected dams compared to mock-infected controls at E18.5, while no such differences were observed at E15.5 or P21 (Fig 2B). These results suggested that transcriptomic changes in our model peak at E18.5 but do not persist into postnatal life. Further analysis revealed thousands of differentially expressed genes (DEGs) in fetal brains harvested at E18.5 (Fig 2C), while we did not detect any statistically significant DEGs at the E15.5 or P21 time points (S2A-S2B Fig). The time point of harvest at E18.5 alone was not the sole determinant of this finding, as similar bulk sequencing analysis on fetal brains collected at E18.5 from dams infected at E16.5 (rather than E12.5) did not yield significant gene expression changes (S3A-S3B Fig). We note that maternal infection at E16.5 also did not result in detectable ZIKV RNA in fetal brains at E18.5 (S3C Fig). Thus, the enrichment of transcriptomic changes in fetal brains at E18.5 following maternal infection at E12.5 is likely driven by a combination of factors, including the presence of ZIKV RNA in fetal brain and the status of maternal infection and antiviral immune responses at this time point.

To characterize specific biological processes likely to be impacted by maternal ZIKV infection, we next performed gene ontology (GO) enrichment analysis of DEGs in E18.5 fetal brain samples. Synaptic function emerged as a dominant theme, as 11 of the top 20 enriched GO terms were associated with synapse-related processes (Fig 2D). A total of 28 synaptic-related terms were enriched across our entire dataset (S4 Fig), including terms such as “Neuron-neuron synaptic transmission” (Fig 2E) and “Regulation of synaptic vesicle clustering” (Fig 2F), suggesting that inapparent maternal ZIKV infection may result in disruptions to synaptogenesis and/or synaptic function even in the absence of overt neurodevelopmental deficits. Strikingly, we did not observe enrichment of any immune-related GO terms in our dataset, and indeed we did not observe significant differences in the expression of any major immune factors in our analysis, including inflammatory chemokines or cytokine signaling molecules commonly induced by flavivirus infection (Fig 2G-2H). These surprising findings suggest that the impact of inapparent ZIKV infection on the fetal brain is not primarily driven by local neuroinflammation, but instead may be driven by neuron-intrinsic responses that impact synaptic biology. Together, these data indicate that maternal ZIKV infection at E12.5 induces transient but substantial transcriptomic dysregulation in fetal brains, characterized by major changes to gene expression related to synaptic neurobiology but not neuroinflammation.

### Single-nucleus RNA sequencing reveals cell-type-specific transcriptomic changes in fetal brains following inapparent maternal ZIKV infection

**Fig 3:**
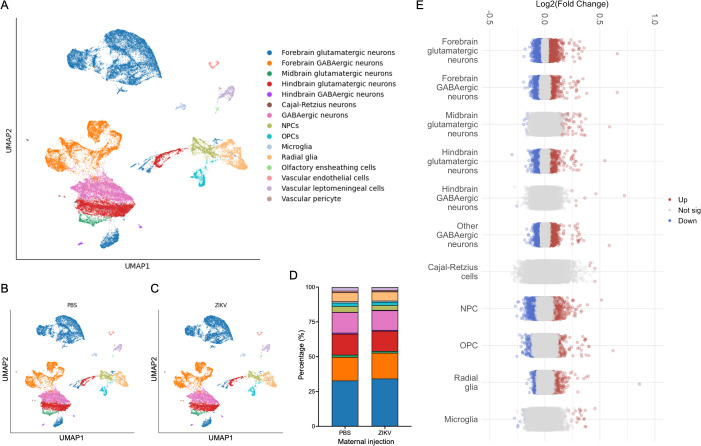
Single-nucleus RNA sequencing reveals cell-type-specific transcriptomic changes in fetal brains following inapparent maternal ZIKV infection. **(A-C)** Uniform manifold approximation and projection (UMAP) analysis of snRNA-seq data from E18.5 fetal brains following maternal infection on E12.5. Panel **(A)** represents the entire dataset, while panel B represents only mock-infected **(B)** or ZIKV-infected **(C)** groups. **(D)** Proportion of individual cell types represented in E18.5 fetal brains in indicated groups. **(E)** Dot plot illustrating significant DEGs within E18.5 fetal brains across distinct cell types. N = 2 samples per group, with each sample representing a barcoded pool of 2 unique animals (4 unique mice per group).

To further dissect the cellular basis of the transcriptomic alterations observed in fetal brains following maternal ZIKV infection at E12.5, we performed single-nucleus RNA sequencing (snRNA-seq) on E18.5 fetal brains derived from infected and control dams. Cell clusters were identified and assigned to specific cell types using established markers (Figs 3A and S5). Fetal brains derived from infected dams exhibited normal frequencies of major brain cell types, and we observed no significant differences in overall cell-type composition between groups (Fig 3B-3D). These data suggest that vertical infection in our model did not result in dropout of major cell types susceptible to ZIKV infection, nor were the lineage commitments of neural stem cells greatly altered at this timepoint, as has been observed in more severe models of congenital ZIKV infection [24,32–34]. However, when we examined DEGs within the major cell types identified in our analysis, we observed robust profiles of differential gene expression across most cell types (Fig 3E). We noted that neural progenitor cells (NPCs) and forebrain glutamatergic neurons (FGNs) displayed particularly pronounced transcriptomic changes in our analysis. This finding prompted us to focus our subsequent analyses on these two cell types to better understand potential cell-type-specific effects of maternal ZIKV infection.

### Maternal ZIKV infection induces cell-type-specific dysregulation of genes related to synapse function and neuronal cell biology

**Fig 4:**
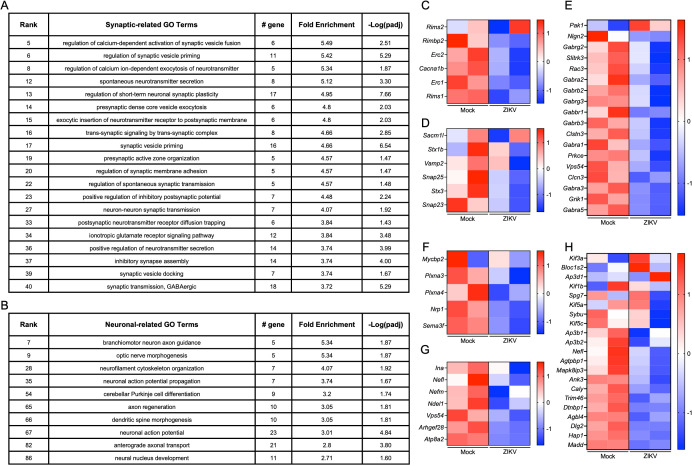
GO enrichment analysis of downregulated DEGs in NPCs reveals terms primarily associated with synaptic function and neuronal cell biology. **(A–B)** Top 20 synaptic-related **(A)** and top 10 neuronal-related **(B)** GO terms among the significantly enriched GO terms obtained from DEGs in NPCs, defined by comparing samples from ZIKV-exposed fetuses to controls in the snRNA-seq dataset shown in Fig 3. **(C-E)** Heatmaps depicting relative expression values (z scores) of genes associated with selected synaptic-related GO terms: regulation of calcium-dependent activation of synaptic vesicle fusion **(C)**, exocytic insertion of neurotransmitter receptor to postsynaptic membrane **(D)**, and GABAergic synaptic transmission **(E)**. **(F-H)** Heatmaps depicting relative expression values of genes associated with selected neuronal-related GO terms: branchiomotor neuron axon guidance **(F)**, neurofilament cytoskeleton organization **(G)**, and anterograde axonal transport **(H)**.

Previous work suggests that ZIKV preferentially infects NPCs in the fetal brain [35–37]. NPCs are essential for neurogenesis and the formation of neural circuits during brain development, and perturbations to their gene expression profiles may incur long-lasting changes to brain function throughout life [38–40]. We therefore first investigated transcriptomic alterations within this critical cell population by performing GO enrichment analyses on DEGs in NPCs derived from infected vs. control fetal brains, analyzing upregulated and downregulated genes separately. Intriguingly, downregulated DEGs in NPCs were significantly enriched for terms associated with synapse function and neuronal cell biology (Fig 4A and 4B), suggesting that maternal ZIKV infection may lead to a reduction in the expression of genes critical for synaptic development and neuronal function within NPCs. Heatmaps depicting representative synaptic and neuronal genes confirmed widespread downregulation of genes associated with these GO terms in NPCs derived from infected fetal brains compared to controls (Fig 4C-4H). In contrast, upregulated DEGs within NPCs were predominantly associated with cellular metabolism and RNA processing (S6 Fig), which may reflect a cellular stress response or shifts in metabolic demands due to infection.

**Fig 5:**
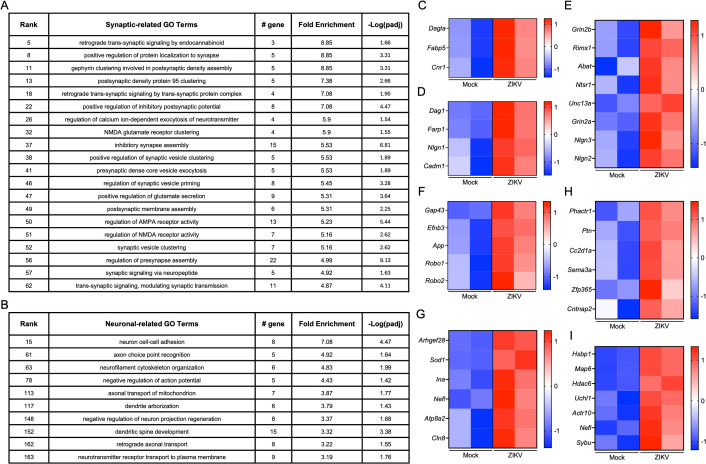
GO enrichment analysis of upregulated DEGs in FGNs reveals terms primarily associated with synaptic function and neuronal cell biology. **(A–B)** Top 20 synaptic-related **(A)** and top 10 neuronal-related **(B)** GO terms among the significantly enriched GO terms obtained from DEGs in FGNs, defined by comparing samples from ZIKV-exposed fetuses to controls in the snRNA-seq dataset shown in Fig 3. **(C-E)** Heatmaps depicting relative expression values (z scores) of genes associated with selected synaptic-related GO terms: retrograde trans-synaptic signaling by endocannabinoids **(C)**, retrograde trans-synaptic signaling by trans-synaptic protein complex **(D)**, and positive regulation of inhibitory postsynaptic potential **(E)**. **(F-H)** Heatmaps depicting relative expression values of genes associated with selected neuronal-related GO terms: axon choice point recognition **(F)**, neurofilament cytoskeleton organization **(G)**, dendrite arborization **(H)**, and axonal transport of mitochondrion **(I)**.

FGNs, another cell type susceptible to ZIKV infection and crucial for excitatory neurotransmission [41], also exhibited robust differential gene expression in our snRNA-seq analysis. Notably, we observed a distinct pattern of transcriptomic changes in FGNs compared to NPCs. While synaptic and neuronal cell biology-related GO terms were significantly enriched among downregulated DEGs in NPCs (Fig 4A-4B), these terms were enriched among the *upregulated* DEGs in FGNs (Fig 5A-5B). Heatmaps of representative synaptic and neuronal genes confirmed robust upregulation of a broad array of genes associated with these terms in FGNs from infected fetal brains (Fig 5C-5I). In contrast, GO enrichment analysis of downregulated DEGs in FGNs revealed terms primarily associated with metabolic processes and RNA splicing (S7 Fig). These findings suggest that maternal ZIKV infection induces cell-type-specific transcriptomic dysregulation, with NPCs exhibiting downregulation of genes related to synaptic function and FGNs exhibiting upregulation of these genes.

**Fig 6:**
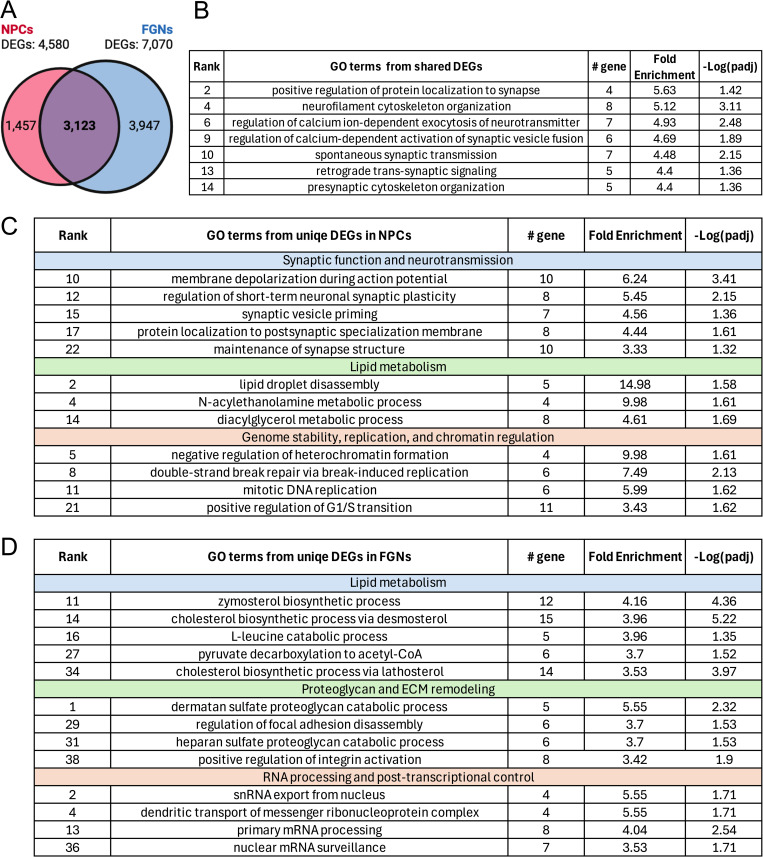
GO enrichment analysis reveals both shared and unique transcriptional pathways altered in NPCs and FGNs in the ZIKV-exposed fetal brain. **(A)** Venn diagram depicting unique and shared DEGs from NPCs and FGNs, defined by comparing samples from ZIKV-exposed fetuses to controls in the snRNA-seq dataset shown in Fig 3. **(B)** Representative significantly enriched GO terms derived from GO enrichment analysis of shared DEGs between NPCs and FGNs. **(C-D)** Representative significantly enriched GO terms derived from GO enrichment analysis of unique DEGs in NPCs **(C)** and FGNs **(D)**. Representative terms in **(C-D)** are further organized into loose themes (color-coded subdivisions) designated post hoc by the authors. Panel A was created in BioRender. Daniels, B. (2026). https://biorender.com/k85vzov.

To further compare how transcriptomic changes are partitioned across these two cell types, we next examined the extent of overlap and divergence in their respective DEG lists. FGNs from ZIKV-exposed embryos exhibited 7,070 DEGs compared to mock-infected controls, while NPCs exhibited 4,580 (Fig 6A). Both cell types shared 3,123 DEGs in common, consistent with our previous analysis revealing similarly enriched GO terms in both. GO enrichment analysis of only this overlapping set of genes again showed significant enrichment of GO terms related to synaptic structure and function (Fig 6B), further confirming that both cell types engage similar transcriptional programs in the ZIKV-exposed fetal brain. We next performed GO enrichment analysis of those DEGs that were uniquely differentially expressed in each cell type. Notably, this analysis revealed that NPCs uniquely exhibited differential expression of genes associated with additional GO terms related to synaptic function and neurotransmission that were not observed in FGNs (Fig 6C), suggesting that modulation of synapses in ZIKV-exposed fetuses occurs through both common and cell type-specific transcriptional programs across neuronal development. NPCs also uniquely exhibited enrichment of GO terms related to genome stability and DNA replication (Fig 6C), likely reflecting their status as highly proliferative cells compared to FGNs, which are postmitotic.

Analysis of GO terms uniquely enriched in FGNs revealed changes to gene expression related to extracellular matrix (ECM) remodeling and RNA processing (Fig 6D). FGN-specific enrichment of proteoglycan metabolic and focal adhesion-related GO terms suggests that maternal ZIKV exposure may selectively alter transcriptional programs linked to ECM organization in excitatory neurons, which may also impact neuronal function by impacting the physical organization of mature dendritic and/or synaptic structures. Both NPCs and FGNs exhibited transcriptional changes associated with lipid metabolism, although specific GO terms differed between cell types, with NPCs showing enrichment for lipid droplet disassembly, N-acylethanolamine metabolism, and diacylglycerol metabolism (Fig 6C), whereas FGNs were enriched for cholesterol/sterol biosynthetic pathways (Fig 6D). This pattern suggests that maternal ZIKV exposure alters transcription associated with lipid metabolism in both cell types, potentially reflecting distinct metabolic demands or stress responses in progenitors versus differentiated neurons, although the functional impact of these changes remains to be determined.

### Maternal ZIKV infection increases hippocampal synaptic density and seizure susceptibility in offspring

**Fig 7:**
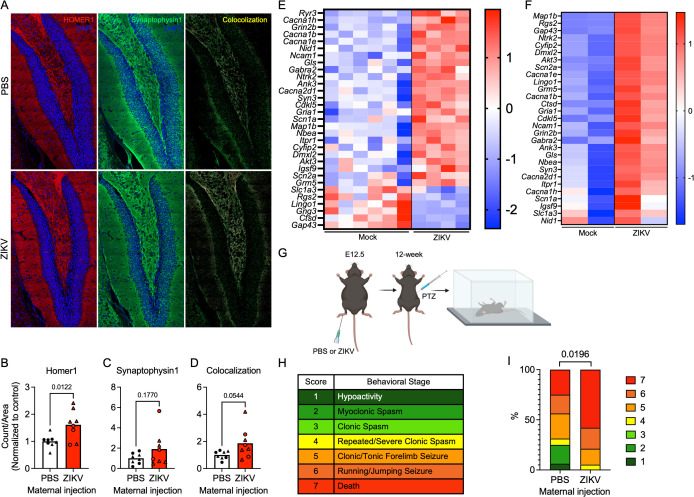
Maternal ZIKV infection increases hippocampal synaptic density and seizure susceptibility in offspring. **(A)** Immunohistochemical (IHC) staining of dentate gyri in P30 offspring brains following maternal infection on E12.5, displaying excitatory postsynaptic HOMER1 (red), pan-presynaptic Synaptophysin1 (green), nuclear DAPI (blue), and colocalization of HOMER1 and Synaptophysin1 (yellow). **(B-D)** Quantification of particle counts of HOMER1 **(B)**, Synaptophysin1 **(C)**, and colocalization **(D)**. N = 8 mice per condition. Male mice are depicted with triangles, while females are depicted with circles. **(E-F)** Heatmaps depicting relative expression values (z scores) of genes associated with seizure in bulk RNA-seq of whole brains **(E)** and snRNA-seq of FGNs **(F)** from E18.5 fetal brains following maternal infection on E12.5. **(G)** Schematic depicting the pentylenetetrazol (PTZ) model of seizure. **(H)** Table explaining the modified Racine Scale of murine seizure stages. **(I)** Portion of mice reaching indicated behavioral seizure stages. n = 16-19 mice per condition. Panel G was created in BioRender. Daniels, B. (2026). https://biorender.com/w5jwemr.

Considering the significant enrichment of synaptic-related GO terms in both our bulk RNA-seq and snRNA-seq datasets, we next investigated whether these transcriptomic alterations were accompanied by morphological changes in the brains of offspring. We performed immunohistochemical (IHC) staining on brains derived from offspring of infected dams at one month following birth (P30) using antibodies against HOMER1, an excitatory postsynaptic marker, and synaptophysin, a presynaptic marker. In the dentate gyrus of the hippocampus, offspring from infected dams exhibited a significant increase in HOMER1-positive puncta compared to controls (Fig 7A-7B), indicating an increase in excitatory postsynaptic structures. While no significant differences were observed in synaptophysin density, we observed an overall increase in the density of colocalized HOMER1 and synaptophysin puncta, which represent functional excitatory synapses (Fig 7A and 7C-7D). These results suggest that the transient transcriptomic changes occurring *in utero* following maternal ZIKV infection were associated with increased abundance of excitatory postsynaptic structures 30 days after birth, when transcriptomic changes have resolved (Fig 2B) and virus is no longer detectable in offspring brains (Fig 1H). We also observed increased immunoreactivity for the astrocyte marker GFAP in hippocampi but not cerebral cortices of P30 offspring exposed to maternal ZIKV infection (S8 Fig), while staining for the myeloid cell marker IBA1 was unchanged in either brain region. The observation of astrogliosis in the absence of any other indicators of neuroinflammation at this time point (including our transcriptomic analysis) suggests that alterations to hippocampal astrocytes may be more related to changes in their neuroregulatory functions.

To determine the potential consequences of these findings, we next sought to determine if offspring in our model exhibited other molecular and functional differences consistent with changes to excitatory synapse density in the forebrain. Clinical reports have indicated that children exposed to ZIKV in utero may experience neurological complications, including seizures, even in the absence of overt microcephaly or other structural abnormalities [42,43]. Given the upregulation of synaptic genes in FGNs (Fig 5A-5D) and the increased abundance of excitatory postsynaptic structures in the postnatal hippocampus (Fig 7A-7B), we hypothesized that inapparent maternal ZIKV infection might lead to dysregulation of genes associated with neuronal excitation and seizure susceptibility. We thus examined both our bulk RNA-seq and snRNA-seq datasets for the expression of seizure-associated genes, which revealed that many genes known to be involved in neuronal excitability and seizure disorders were significantly dysregulated in offspring from infected dams compared to controls in both whole brains (Fig 7E) and specifically within FGNs (Fig 7F). Notably, seizure-associated genes were predominantly upregulated in both datasets, which, along with our imaging findings, suggested that offspring of infected dams may exhibit increased susceptibility to seizure. To assess this, we evaluated seizure susceptibility in 12-week-old offspring using the pentylenetetrazol (PTZ) seizure model. PTZ is a GABA-A receptor antagonist commonly used to induce seizures in rodents by inhibiting inhibitory neurotransmission [44]. Offspring from ZIKV-infected and control dams were administered PTZ, and seizure activity was recorded and scored using a modified Racine scale (Fig 7G-7H). Notably, offspring from infected dams exhibited significantly higher seizure sensitivity and severity, including seizure-induced death, compared to controls (Fig 7I). These findings indicate that the synaptic alterations and gene expression changes induced by inapparent maternal ZIKV infection have functional consequences on neuronal excitability, including increased susceptibility to seizure.

### Motion sequencing reveals altered spontaneous behavioral structure in adult offspring exposed to maternal ZIKV infection

**Fig 8:**
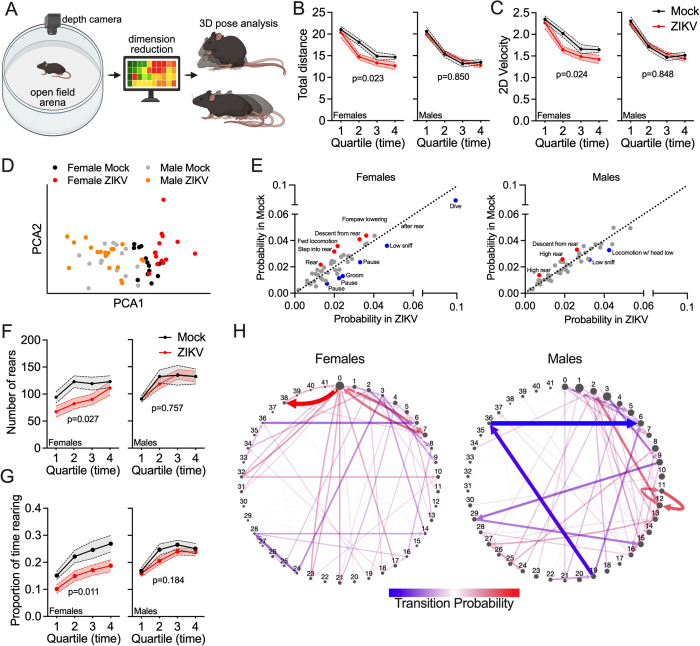
Motion Sequencing (MoSeq) reveals altered spontaneous behavioral structure in adult offspring exposed to maternal ZIKV infection. **(A)** Schematic overview of MoSeq (Motion Sequencing) experimental workflow. Offspring from ZIKV-infected and control dams were recorded in an open field arena and 3D behavior was analyzed using unsupervised machine learning to identify discrete syllables of motor activity. **(B–C)** Total distance traveled **(B)** and 2D velocity profiles **(C)** over 20 minutes of spontaneous activity in the open field, split into 5 minute quartiles. **(D)** Principal component analysis (PCA) of MoSeq behavioral syllable usage across all offspring, grouped by sex and maternal infection status. **(E)** Scatterplots depicting individual syllable usage probabilities. Syllables used more frequently by control animals are labeled in red; those used more frequently by ZIKV-exposed animals are labeled in blue. **(F–G)** Quantification of rearing behavior during the 20-minute open field trial, shown as total number of rears **(F)** and cumulative rearing duration **(G)**. **(H)** Transition probability plots highlighting behavioral syllable transitions that occur at different frequencies between groups. Each line represents a syllable-to-syllable transition with a notable group difference; line thickness indicates the absolute magnitude of difference in probability between groups, and color denotes whether a given transition is observed more (red) or less (blue) frequently in ZIKV-exposed animals. N = 13-20 mice/sex/group in all comparisons. Panel A was created in BioRender. Daniels, B. (2026). https://biorender.com/uqc8vcu.

To further characterize the long-term behavioral effects of inapparent maternal ZIKV infection, we employed motion sequencing (MoSeq), an unsupervised machine learning platform that captures spontaneous behavior by recording depth video of freely moving animals and segmenting behavior into discrete sub-second motifs, or “syllables,” based on 3D pose dynamics (Fig 8A). This approach enables high-resolution quantification of both the frequency of individual behavioral syllables and the structure of transitions between them, allowing for detection of subtle disruptions in behavioral organization that may not be evident through traditional analyses. Analysis of open field performance revealed sex-specific effects of maternal ZIKV infection, as adult female offspring from ZIKV-infected dams exhibited significantly reduced total distance traveled and lower locomotor velocity across the trial, whereas adult male offspring showed no change from controls (Fig 8B–8C). Principal component analysis of syllable usage patterns showed that ZIKV-exposed females formed a distinct behavioral cluster, suggesting that maternal infection induces a unique alteration in the structure of spontaneous behavior in female offspring (Fig 8D). Syllable usage analysis revealed a shift away from rearing-associated syllables toward those associated with pausing and diving behaviors, a pattern observed more strongly in females but also weakly in males (Fig 8E). To validate this shift, we quantified rearing behavior using syllable-based annotations and found that ZIKV-exposed females displayed a significant reduction in both the number and cumulative duration of rears, with no significant changes in males (Fig 8F–8G). Despite these sex differences in syllable usage, analysis of syllable-to-syllable transition probabilities revealed alterations in behavioral sequencing in both sexes, with specific transitions occurring more or less frequently in ZIKV-exposed animals (Fig 8H). We note that sexually dimorphic behavioral phenotypes in adults did not appear to be correlated with obvious sex differences in gene expression at E18.5, as only a small number of DEGs in our RNA-seq analysis showed significant divergence between sexes at this time point (S9 Fig). Together, these data indicate that inapparent maternal ZIKV infection leads to persistent, sex-biased changes in spontaneous behavioral structure, with pronounced effects on both the content and organization of behavior in female offspring. These behavioral changes likely arise through complex processes that play out across development, including sexual maturation.

## Discussion

Our findings provide much needed new insight into the potential long-term outcomes associated with in utero ZIKV exposure, beyond the well-characterized impacts of CZS. While severe outcomes such as microcephaly have been linked to maternal infection during early gestation [2,3], less is known about the subtler effects of ZIKV exposure during mid to late gestation. Studies in both humans and animal models suggest that the timing of infection critically influences fetal development, with early gestational exposure leading to more pronounced structural abnormalities and later exposure potentially causing functional deficits without obvious morphological irregularities [6,21,45,46]. Our mouse model of maternal infection at mid-gestation in immunocompetent *hSTAT2* mice thus provides a valuable platform to investigate these subtler neurodevelopmental impacts. By more faithfully modeling inapparent maternal infection in humans, our model will facilitate the study of how mild ZIKV exposure during different gestational stages affects long-term neurological outcomes in offspring.

Our single-nucleus RNA sequencing dataset also provides a high-resolution atlas of cell type-specific transcriptomic changes in the fetal brain following inapparent maternal ZIKV infection. Unlike existing studies that often focus on severe infection models or utilize bulk RNA sequencing without cellular resolution [21,45,47], our dataset captures nuanced transcriptomic alterations across diverse neuronal populations during late gestation, a critical period for brain development. Beyond NPCs and FGNs, our dataset provides detailed profiles of other neuronal subtypes, such as GABAergic interneurons and neurons in distinct regions like the hindbrain. GABAergic interneurons are essential for maintaining excitatory-inhibitory balance in neural circuits, and disruptions in their development have been linked to neurodevelopmental disorders like autism spectrum disorder (ASD) and epilepsy [48–51]. Similarly, hindbrain neurons play crucial roles in autonomic functions and motor control [52–54]; alterations in these neurons can also lead to disorders affecting movement and physiological regulation [55]. Studies have shown that fetal exposure to pathogens can impact the development of these neuronal populations, potentially resulting in long-term neurological deficits [56–60]. By providing this comprehensive resource, our dataset will enable others in the field to explore how inapparent maternal ZIKV infection affects the development and function of diverse neuronal subtypes.

The observed increase in synaptic density and excitability in the brains of offspring from ZIKV-infected dams in our study also raises important considerations regarding links between fetal ZIKV exposure and risk for neurodevelopmental disorders such as ASD. Enhanced excitatory synaptic connectivity has been implicated in the pathophysiology of ASD and other conditions characterized by altered neuronal circuitry and excitability [61,62]. Our findings suggest that even mild maternal ZIKV infection can lead to synaptic alterations that may predispose offspring to such outcomes. Currently, children exposed to ZIKV in utero but without CZS are not routinely identified or monitored for subsequent neurodevelopmental issues. Our study underscores the need for improved surveillance and follow-up in these cases, which represent the vast majority of human ZIKV infections during pregnancy.

In addition to effects on synaptic density and seizure susceptibility, our MoSeq analysis revealed persistent alterations in spontaneous behavior following inapparent maternal ZIKV infection, particularly in female offspring. The observed reductions in open field activity, rearing behavior, and shifts in syllable usage suggest that ZIKV exposure may disrupt the maturation of exploratory and motor control circuits during late gestation. Moreover, changes in syllable transition structure in both sexes indicate that maternal infection may alter the temporal organization of spontaneous behavior, even in the absence of gross motor deficits. These findings reinforce the sensitivity of behavioral sequencing as a functional readout of early-life neural disruption and support the use of MoSeq as a tool for identifying latent phenotypes associated with prenatal viral exposure. Together with our transcriptomic and histological data, these behavioral results suggest that even transient fetal brain infection can lead to durable alterations in neural circuit function, with sex-specific consequences that may shape long-term neurodevelopmental trajectories.

Our findings build on a growing body of research suggesting that in utero exposure to ZIKV can result in subtle but consequential perturbations to brain health and behavior, even in the absence of CZS. While limited, the existing clinical literature has described a diverse array of neurologic sequalae in normocephalic children exposed to ZIKV, including memory and learning impairments [63], motor coordination difficulties [64,65], and issues with language development [66,67]. Neuroimaging studies also reveal that ZIKV-exposed children without structural abnormalities at birth may nevertheless develop structural pathologies in the first years of life [64,68]. These risks are also likely influenced by the biological sex of affected infants [69]. In our study, female offspring of ZIKV-infected dams experienced more pronounced changes to spontaneous locomotor behavior compared to male littermate controls. These findings are consistent with other studies in mouse models showing more pronounced behavioral deficits in female animals exposed to ZIKV in utero [70], although how such findings may extend to human children remains unclear. Collectively, these findings further underscore the need for enhanced investment and attention to the long-term health trajectories of children exposed to ZIKV.

Our observation of increased excitatory postsynaptic structures in the hippocampus, heightened susceptibility to PTZ-induced seizures, and alterations in spontaneous behavioral structure in ZIKV-exposed offspring also suggests broad changes to excitatory neurotransmission that may map onto clinically observable phenotypes in humans. Early life screening in ZIKV-exposed children using approaches such as EEG assessment of cortical excitability and excitatory/inhibitory (E/I) balance could serve as targets for testing mechanism-based interventions. Research using EEG-based screening shows immense potential for early detection of neurological disorders such as ASD [71]. Altered cortical excitability is also observed in schizophrenia and bipolar disorder [72], and disruption to E/I balance is implicated in disorders such as AD [73]. Animal models of maternal immune activation (MIA) have also demonstrated lasting reductions in EEG coherence and E/I balance that persist into adulthood [74,75]. Notably, pharmacological and neuromodulatory interventions have been shown to ameliorate abnormal EEG features in several neurological disorders [76], and thus existing therapies that target abnormal neural excitation may merit mechanistic study in the context of in utero ZIKV exposure.

Our data also indicate that during clinically inapparent maternal ZIKV infection, fetal brains exhibited dysregulation of synapse-related pathways without engagement of immunological gene programs, suggesting that neurodevelopmental abnormalities may have arisen from neuron-intrinsic mechanisms rather than neuroinflammation. However, we cannot absolutely exclude the possibility that an earlier, transient innate immune response contributed to our observed phenotypes. Indeed, maternal tissues exhibited substantial cytokine induction, and MIA is widely known to influence synaptic gene expression, receptor trafficking, dendritic growth, and microglia-mediated pruning in offspring [77,78]. Such signals can act over short windows yet produce durable shifts in circuit assembly. However, we note that infection at E16.5, which did not yield detectable fetal brain infection, failed to produce transcriptomic changes in fetal brain at E18.5 or behavioral phenotypes postnatally, suggesting that maternal immune activation alone is unlikely to account for the outcomes we observe after infection at E12.5. Further mechanistic study of maternal vs. fetal determinants of neurologic outcome in ZIKV-exposed offspring will be needed.

An important additional consideration in the interpretation of our findings is how the time points of analysis in our mouse model map onto the timeline of human gestation and development. Maternal infection at E12.5 in mice corresponds approximately to late first-trimester stages of human gestation [79], when cortical neurogenesis is robust and radial glia–like NPCs are actively expanding and differentiating into early-born projection neurons [80,81]. Comparative analyses of rodent and human corticogenesis indicate that this window encompasses the peak production of deep and mid-layer forebrain glutamatergic neurons [82,83], including early circuit assembly [84,85]. By E18.5, when we observe pronounced transcriptomic changes in NPCs and FGNs, the mouse brain is in late gestation, roughly analogous to late second to early third trimester in humans [86,87], a period characterized by waning progenitor proliferation, continued differentiation of glutamatergic neurons, and the onset of synaptogenesis and dendritic elaboration [88–90]. Our observation that synaptic and neuronal genes are decreased in NPCs but increased in FGNs at this time point suggests that inapparent maternal ZIKV infection does not simply strengthen or weaken a single synaptic program across the fetal brain. One possible interpretation is that progenitors in ZIKV-exposed offspring reduce expression of genes that normally prepare them for neuronal and synaptic maturation, while already differentiated glutamatergic neurons shift toward a more synaptically mature profile than expected for this age. Thus, ZIKV exposure may disturb the normal temporal coordination between progenitor state and neuronal maturation, which could change how cortical and hippocampal circuits are assembled even without causing gross loss of specific cell types.

Similarly, the postnatal readouts in our model align with developmental stages in humans that extend into late gestation and early childhood. In mice, the first two postnatal weeks are often considered broadly equivalent to the human third trimester [91,92], which features intense synaptogenesis, spine formation, and circuit refinement in hippocampus and cortex [91,93,94]. The P21–P30 time points at which we assess hippocampal synaptic density fall just after this peak and are roughly comparable to the human early infancy period [95,96]. The MoSeq and seizure assays performed in 12–20 week old mice thus examine the long-term functional consequences of mid-gestational infection and transient fetal brain infection on circuits that, in humans, would be maturing from late gestation through the early postnatal years. Framing our results in this comparative context suggests that mild ZIKV infection during human mid-gestation could perturb NPC and FGN programs during a critical window for cortical and hippocampal circuit assembly, with consequences that manifest later in childhood and adolescence as altered excitability and spontaneous behavioral structure, even in the absence of overt structural malformations at birth.

We note that our study uses an African lineage ZIKV strain (DAKAR) which was adapted to mice for use in tandem with the *hSTAT2* knockin mouse line. This mouse adapted strain differs from its wildtype parent strain by only two (non-synonymous) amino acid mutations, of which only the G18R mutation in the NS4B protein appears to functionally confer enhanced pathogenesis in mice [25]. Comparison of ZIKV strains harboring this mutation (versus isogenic controls) showed that *hSTAT2* mice exhibit increased ZIKV replication in both brain and lymphoid tissues following infection, suggesting that the NS4B G18R mutation enhances pathogenicity through broad, rather than brain-specific, mechanisms of murine-specific host restriction. Thus, we believe that our findings using this strain are unlikely to arise as artefacts of mouse adaptation or enhanced neurovirulence. Nevertheless, future studies should also explore differential outcomes following infection with divergent strains of ZIKV. Prior work has suggested that Asian lineage ZIKV strains may be associated with more severe fetal outcomes compared to African strains [97,98], although recent evidence has called this association into question [99–101]. Moreover, while strain differences have been explored in the context of CZS [101–103], their impact on subtler neurodevelopmental outcomes remains underinvestigated. Differences in viral genetics could influence pathogenicity even in the setting of inapparent infection, potentially leading to varying effects on fetal brain development. Additionally, maternal immune factors and fetal susceptibility may differentially interact with distinct ZIKV strains. Understanding these nuances will require studies that delineate the contributions of both maternal and fetal responses to major ZIKV lineages. Such research is crucial for developing comprehensive models of ZIKV pathogenesis and for informing public health strategies tailored to specific geographic and epidemiological contexts.

In conclusion, our study highlights potentially significant but overlooked impacts of inapparent maternal ZIKV infection on fetal brain development and long-term neurological function. As climate change expands the geographic range of mosquito vectors, the threat of ZIKV and other emerging flaviviruses persists and may intensify [104–106]. Our findings emphasize the necessity for heightened attention to these viruses, including enhanced surveillance, research efforts, and public health initiatives. By providing a relevant animal model and new transcriptomic datasets, we hope to stimulate fresh attention to the study of ZIKV infections during pregnancy in order to expand our ability to understand and address the extensive public health burden posed by ZIKV and similar pathogens.

## Methods and materials

### Ethics statement

All animal work in this study was done with the approval of the Rutgers University Institutional Animal Care and Use Committee (Protocol Number: 201900016).

### Viruses

ZIKV-DAKAR-MA was first generated [25] and was generously provided by Dr. Michael Diamond (Washington University, St. Louis, MO, USA). Viral stocks were generated by infecting Vero E6 cells (ATCC #CRL-1586) (MOI 0.01) and harvesting supernatants at 72hpi. Stock titers were determined via plaque assay on Vero E6 cells. Cells were maintained in DMEM (Corning #10–013-CV) supplemented with 10% Heat Inactivated FBS (Gemini Biosciences #100–106), 1% Penicillin-Streptomycin-Glutamine (Gemini Biosciences #400–110), 1% Amphotericin B (Gemini Biosciences #400–104), 1% Non-Essential Amino Acids (Cytiva #SH30238.01), and 1% HEPES (Cytiva SH30237.01). Plaque assay basal media was 10X EMEM (Lonza #12-684F) adjusted to 1X and supplemented with 2% Heat Inactivated FBS (Gemini Biosciences #100–106), 1% Penicillin-Streptomycin-Glutamine (Gemini Biosciences #400–110), 1% Amphotericin B (Gemini Biosciences #400–104), 1% Non-Essential Amino Acids (Cytiva #SH30238.01), 1% HEPES (Cytiva SH30237.01), 0.75% Sodium Bicarbonate (VWR #BDH9280) and 0.5% Methyl Cellulose (VWR #K390). Plaque assays were read 4dpi by removal of overlay media and staining/fixation using 10% neutral buffered formalin (VWR #89370) and 0.25% crystal violet (VWR #0528).

### Murine model of ZIKV infection

*hSTAT2* knockin mice were originally obtained from Jackson Laboratories (strain 031630) and subsequently bred in-house. All animals were housed under pathogen-free conditions in the animal facilities in Nelson Biological Laboratories at Rutgers University. Mice were injected subcutaneously (50 μl) in a rear footpad with 10^5^ PFU ZIKV-DAK-MA, as described previously [25].

### Tissue harvesting

Isoflurane anesthesia was used for all procedures. Cardiac blood was collected to test for viral burden in serum. For pregnant female mice, embryos were removed prior to cardiac perfusions with 30 mL cold 1X phosphate-buffered saline (PBS). For quantitative real-time PCR (qPCR), spleens and brains was harvested and homogenized using 1.0 mm diameter zirconia/silica beads (Biospec Products, #11079110z) in TRI Reagent (Zymo, #R2050–1). Homogenization was performed in an Omni Beadrupter Elite for 2 sequential cycles of 20 s at a speed of 4 m/s. For immunohistochemistry (IHC), brains of offspring at P30 were harvested following cardiac perfusion with cold PBS followed by 4% paraformaldehyde (PFA). Perfused brains were stored in 4% PFA overnight followed by PBS at 4°C.

### Quantitative real-time PCR

Total RNA from harvested tissues was isolated with the Zymo Direct-zol RNA Miniprep kit (Zymo #R2051) following the manufacturer’s protocol. Viral RNA from serum was isolated with TRI Reagent (SIGMA-ALDRICH #T3934) following the manufacturer’s protocol. RNA concentration was measured using a Quick Drop device (Molecular Devices). cDNA synthesis and qPCR were performed as previously described [107]. Cycle threshold (CT) values for analyzed genes were normalized to CT values of the housekeeping gene 18S (CT_Target_ – CT_18S_ = ΔCT). Data were further normalized to baseline control values (ΔCT_experimental_ – ΔCT_control_ = ΔΔCT (DDCT)). A list of primers used in this study can be found in S1 Table.

### Seizure assay

Seizures were induced by a single i.p. injection of pentylenetetrazole (PTZ) (Sigma-Aldrich, 54-95-5) at a dose of 60 μg/g of body weight. Behavioral responses were evaluated using a modified version of the Racine scale as previously described [41,108]. Video recordings of mouse seizures were scored by an operator blinded to the experimental condition of each subject.

### Motion sequencing (MoSeq)

Spontaneous behavior was recorded and analyzed using MoSeq, an unsupervised machine learning framework for behavioral profiling, as previously described [109]. Briefly, 12 week old mice were placed individually in an open-field arena and recorded for 20 minutes using a Kinect v2 depth camera positioned overhead. Depth video was processed to extract pose dynamics. Videos were then embedded using principal component analysis (PCA), and the first 10 principal components were modeled using an autoregressive hidden Markov model (AR-HMM) to identify discrete, recurring behavioral “syllables.” Syllable usage frequencies were normalized and compared between groups using bootstrap resampling with Benjamini–Hochberg correction for multiple comparisons.

### Bulk RNA sequencing

Pregnant *hSTAT2* mice were injected subcutaneously with ZIKV at embryonic day (E) 12.5 and brains of offspring were harvested at E15.5, E18.5, and P 21. Brains were homogenized in Trizol and RNA was extracted with Zymo Direct-zol RNA Miniprep kit (Zymo #R2051) following the manufacturer’s protocol. RNA library preparation, sequencing, and preliminary analysis was performed as previously described [41] by Azenta Life Sciences. Bulk RNA sequencing datasets are available through the NCBI Gene Expression Omnibus, accession number GSE302948.

### Single-nucleus RNA sequencing

Pregnant *hSTAT2* mice were injected subcutaneously with ZIKV at E12.5 and fetal brains were harvested at E18.5. Tissue fixation and dissociation were performed using the Chromium Next GEM Single Cell Fixed RNA Sample Preparation Kit (10X Genomics #1000414) and following the manufacturer’s protocol. 10X Genomics Chromium 3’ gene express 10X Genomics Chromium Gixed RNA-seq library preparation, 10X library sequencing through Illumina NovaSeq X Plus, and pre-processing through Cell Ranger were performed by Azenta Life Sciences. Further data normalization, processing, and analysis were performed with Scanpy. Single-nucleus sequencing datasets are available through the NCBI Gene Expression Omnibus, accession number GSE303736.

### Immunohistochemistry

Fixed brains were embedded in agar and sectioned by Compresstome (Precisionary #VF-510-0Z). Sections were blocked with 10% goat serum (Gibco #16210) and 0.4% Triton-X for 3 hours at room temperature, followed by incubation in primary antibody for 48 hours at 4°C. Primary antibodies used were rabbit anti-Homer1 1:200 (Synaptic Systems, 160–003), guinea pig anti-Synaptophysin1 1:250 (Synaptic Systems, 101–004), rat anti-GFAP 1:250 (Invitrogen, 13–0300), and chicken anti-IBA1 1:1000 (Synaptic Systems, 234–009). Sections were washed and incubated in secondary antibodies for 1 hour at room temperature. Sections were stained with DAPI prior to mounting with ProLong Diamond AntiFade (ThermoFisher, P36970).

### Statistical analysis

Statistical analyses were performed using GraphPad Prism 9. Seizure assay was compared via chi-square test. Most other experiments were compared with appropriate parametric tests, including the Student’s t-test (two-tailed) or two-way analysis of variance (ANOVA) with Tukey’s post hoc test to identify significant differences between groups. A p-value of less than 0.05 was deemed to indicate statistical significance. Unless specified otherwise, all data points represent biological replicates consisting of distinct mice.

## Supporting information

S1 Fig Inflammatory cytokines and chemokines in the spleens of infected dams. (A-C) Expression profiles of Cxcl1 (A), Ifna (B) and Tnfa (C) in spleens from pregnant dams and age matched non-pregnant female controls, as assessed by RT-qPCR analysis on 3 and 6 dpi. * p < 0.05, ** p < 0.01, *** p < 0.001.
(PDF)

S2 Fig No DEGs were statistically significant at E15.5 or P21 time points. (A-B) Volcano plot illustrating differential expression of transcripts in E15.5 fetal brains (A) and P21 offspring brains (B) following maternal infection on E12.5.
(PDF)

S3 Fig E18.5 fetal brains following maternal ZIKV infection at E16.5 showed no significant transcriptomic changes. (A) Principal component analysis (PCA) derived from bulk RNA-seq of offspring brains harvested at E18.5 following maternal infection on E16.5. N = 6 mice per condition. (B) Volcano plot illustrating differential expression of transcripts in E18.5 fetal brains following maternal infection on E16.5. (C) Abundance of viral RNA detected in placenta and fetal brains harvested at E18.5 following maternal infection on E16.5, measured by RT-qPCR. Dashed line indicates the limit of detection. N = 18 mice per group.
(PDF)

S4 Fig Synaptic-related GO terms were enriched in E18.5 fetal brains following maternal ZIKV infection on E12.5. (PDF)

S5 Fig Specific cell types were assigned using established markers. (A-B) Markers used to assign neuron (A) and non-neuron (B) cell types in single nucleus RNA sequencing data depicted in Fig 3.
(PDF)

S6 Fig GO enrichment analysis of *up*regulated DEGs in NPCs revealed terms primarily associated with metabolic processes and RNA splicing. (PDF)

S7 Fig GO enrichment analysis of *down*regulated DEGs in FGNs revealed terms primarily associated with metabolic processes and RNA splicing. (PDF)

S8 Fig Maternal ZIKV infection induces hippocampal astrogliosis in offspring. (A) Immunohistochemical (IHC) staining of hippocampi in P30 offspring brains following maternal infection on E12.5, displaying GFAP (magenta), IBA1 (yellow), nuclear DAPI (cyan). (B-C) Quantification of image area positive for GFAP (B) or IBA1 (C). (E) IHC analysis of cerebral cortex using markers and experimental groups described in (A). (E-F) Quantification of image area positive for GFAP (E) or IBA1 (F). * p < 0.05.
(PDF)

S9 Fig DEGs in fetal brains at E18.5 that differ significantly between sexes in ZIKV-exposed fetuses. Heatmap depicting DEGs from analysis of bulk sequencing data described in Fig 2. Depicted genes are significantly altered in ZIKV-exposed fetuses of both sexes compared to mock controls, while also differing significantly between sexes within the ZIKV-exposed group.
(PDF)

S1 Table List of primers used for RT-qPCR. (PDF)
