# Supplementary figures and images for: Inapparent maternal ZIKV infection impacts fetal brain development and postnatal behavior
Source: PLoS Pathog. 2026 Jan 12;22(1):e1013850. doi: 10.1371/journal.ppat.1013850 (PMC12822987; doi:10.1371/journal.ppat.1013850)

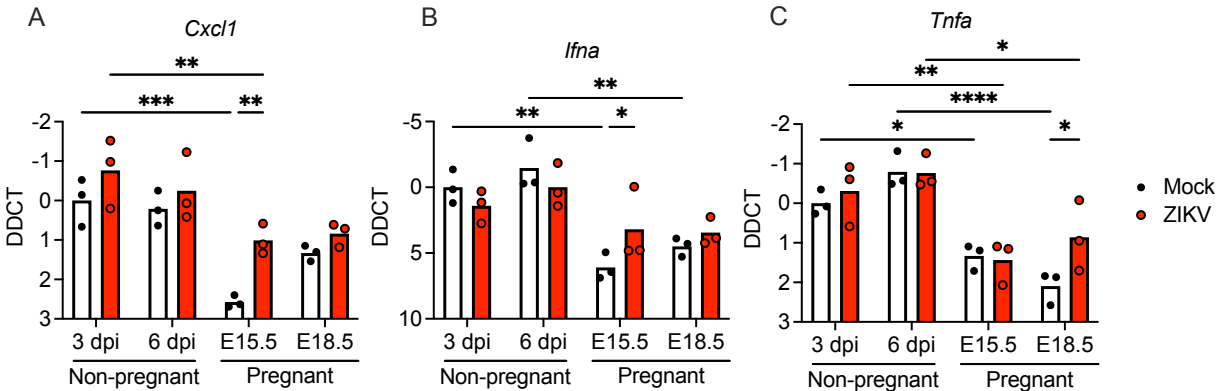

Supplement: S1 Fig — (A-C) Expression profiles of Cxcl1 (A), Ifna (B) and Tnfa (C) in spleens from pregnant dams and age matched non-pregnant female controls, as assessed by RT-qPCR analysis on 3 and 6 dpi. * p < 0.05, ** p < 0.01, *** p < 0.001. (PDF) [file ppat.1013850.s001.pdf]

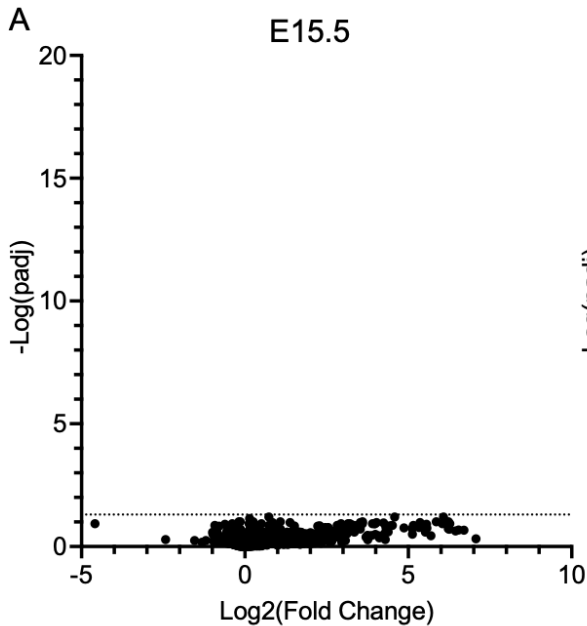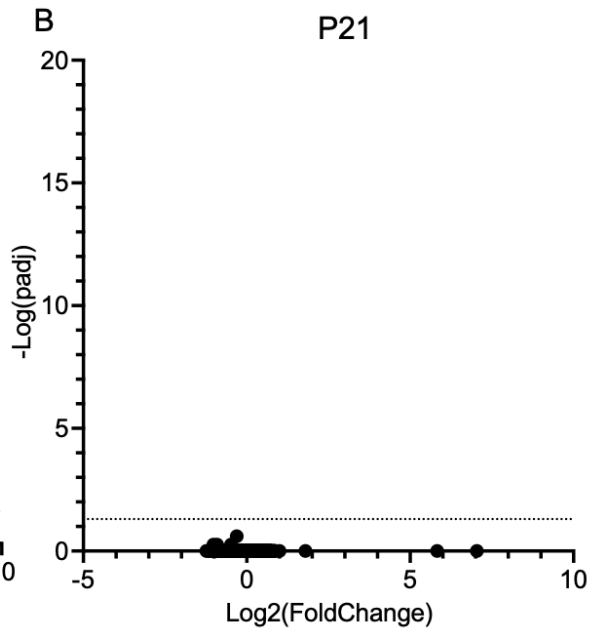

Supplement: S2 Fig — (A-B) Volcano plot illustrating differential expression of transcripts in E15.5 fetal brains (A) and P21 offspring brains (B) following maternal infection on E12.5. (PDF) [file ppat.1013850.s002.pdf]

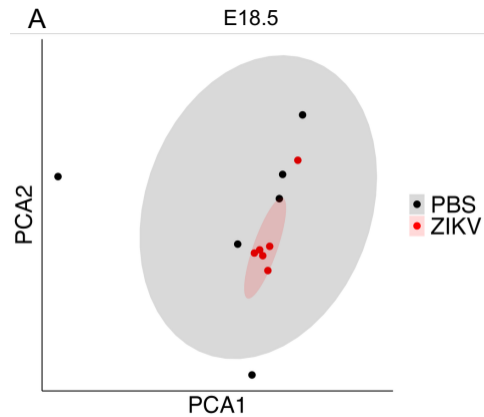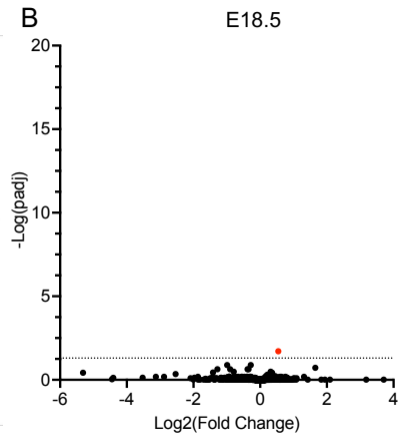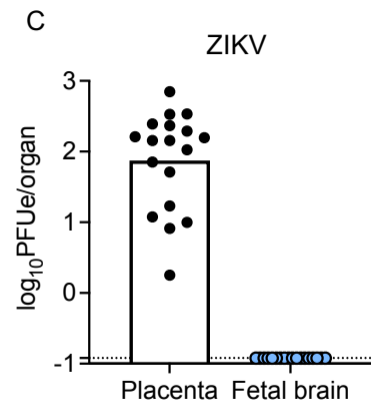

Supplement: S3 Fig — (A) Principal component analysis (PCA) derived from bulk RNA-seq of offspring brains harvested at E18.5 following maternal infection on E16.5. N = 6 mice per condition. (B) Volcano plot illustrating differential expression of transcripts in E18.5 fetal brains following maternal infection on E16.5. (C) Abundance of viral RNA detected in placenta and fetal brains harvested at E18.5 following maternal infection on E16.5, measured by RT-qPCR. Dashed line indicates the limit of detection. N = 18 mice per group. (PDF) [file ppat.1013850.s003.pdf]

A

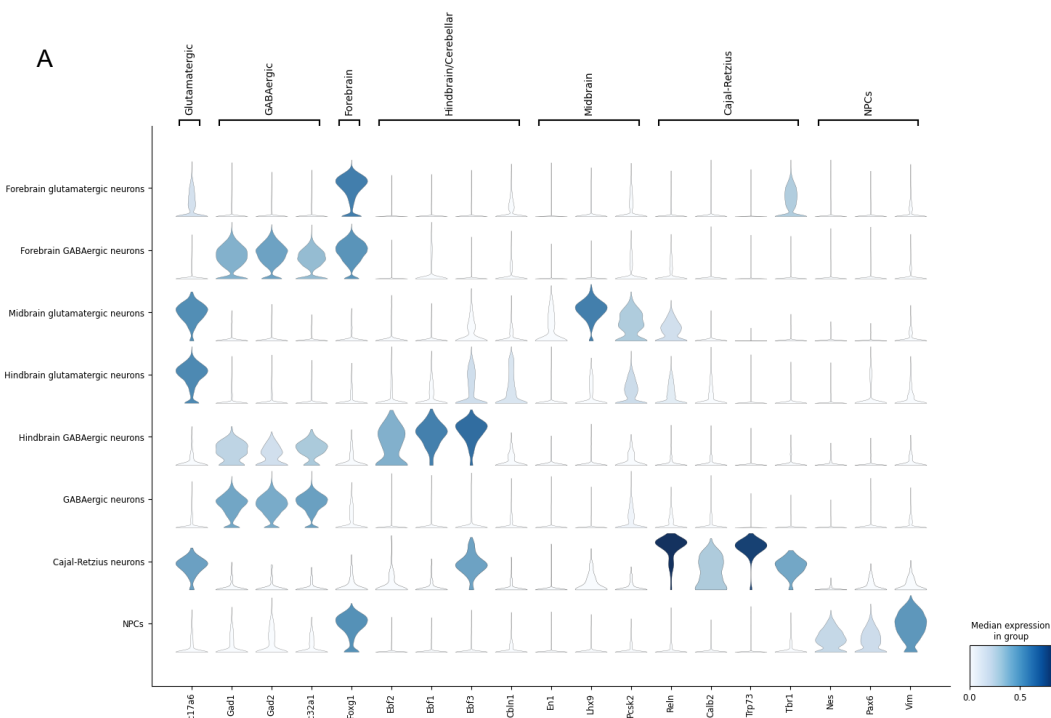

B

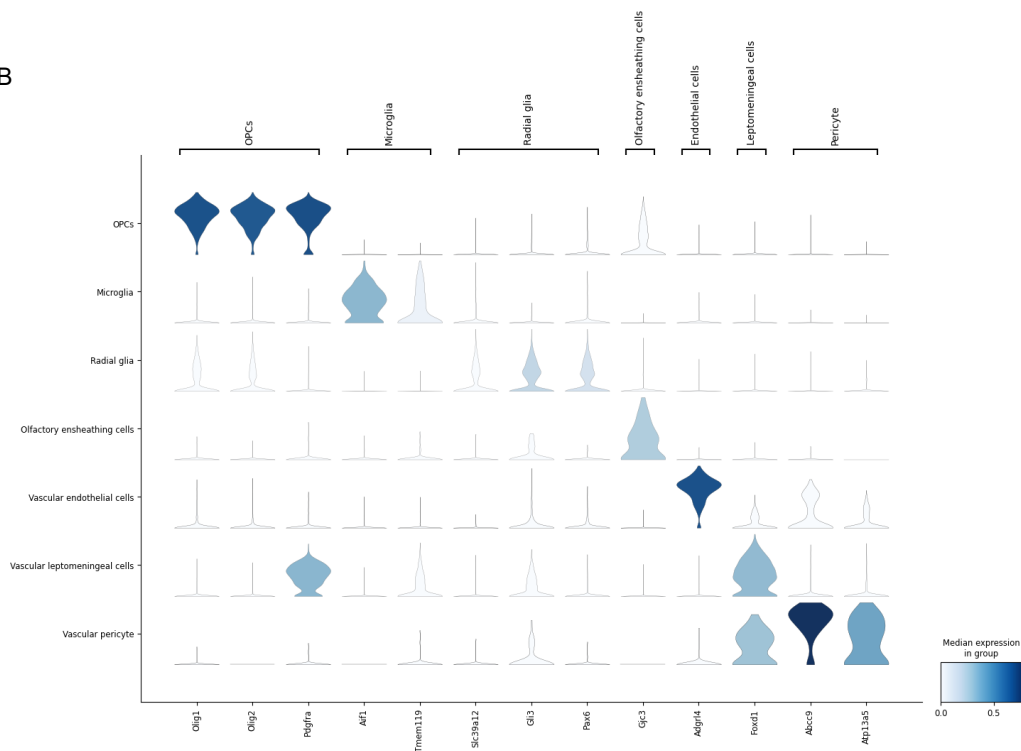

Supplement: S5 Fig — (A-B) Markers used to assign neuron (A) and non-neuron (B) cell types in single nucleus RNA sequencing data depicted in Fig 3. (PDF) [file ppat.1013850.s005.pdf]

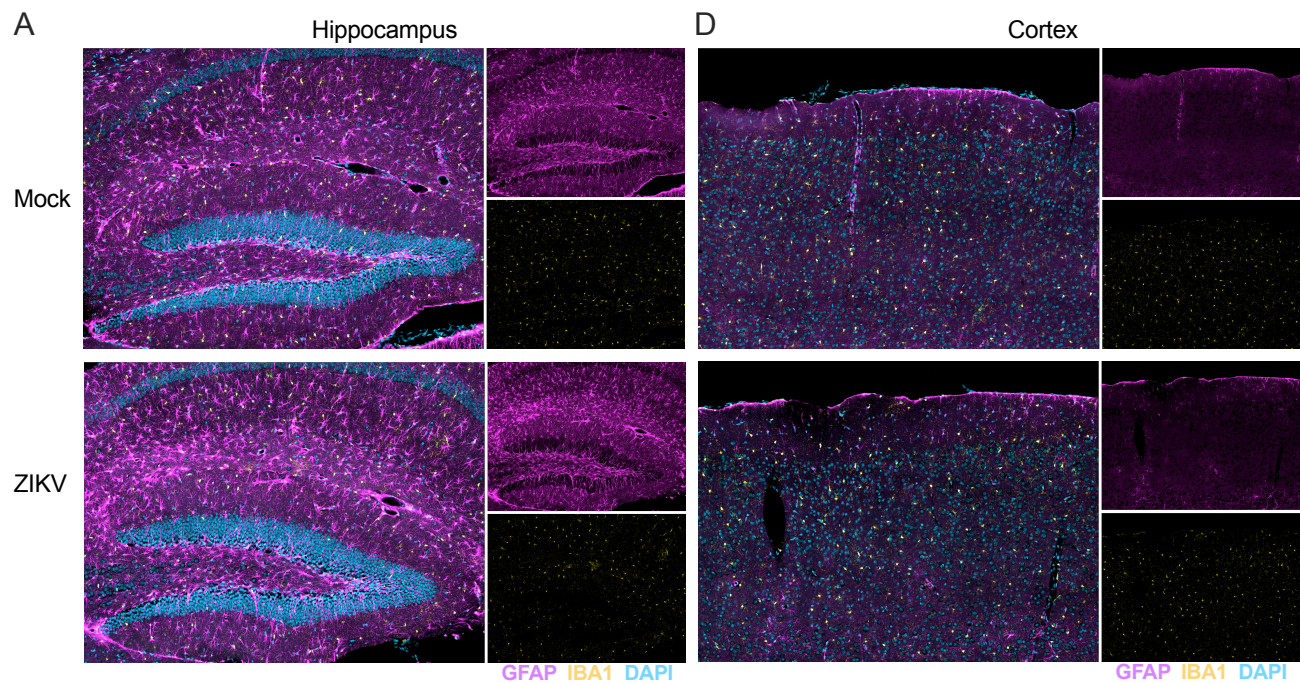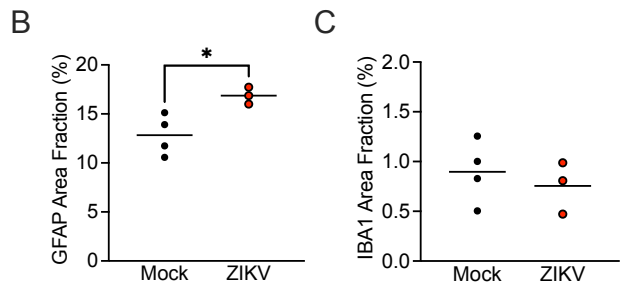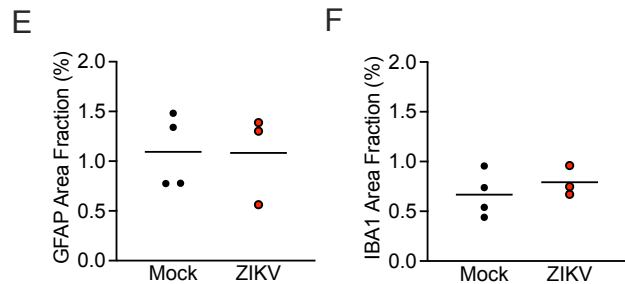

Supplement: S8 Fig — (A) Immunohistochemical (IHC) staining of hippocampi in P30 offspring brains following maternal infection on E12.5, displaying GFAP (magenta), IBA1 (yellow), nuclear DAPI (cyan). (B-C) Quantification of image area positive for GFAP (B) or IBA1 (C). (E) IHC analysis of cerebral cortex using markers and experimental groups described in (A). (E-F) Quantification of image area positive for GFAP (E) or IBA1 (F). * p < 0.05. (PDF) [file ppat.1013850.s008.pdf]

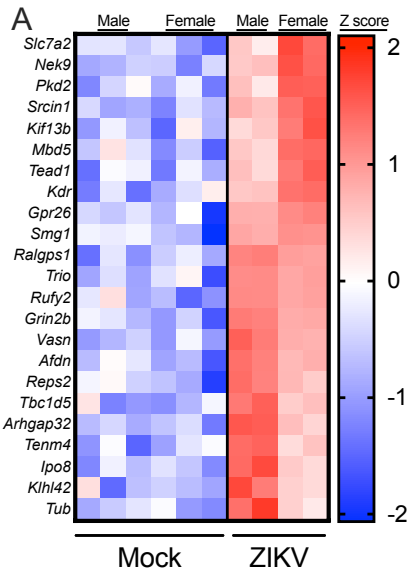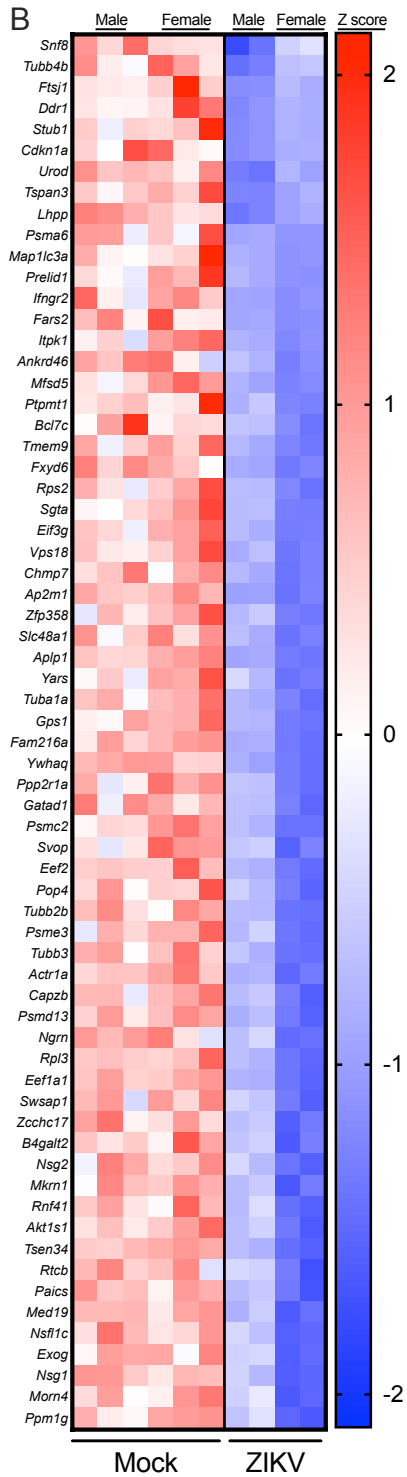

Supplement: S9 Fig — Heatmap depicting DEGs from analysis of bulk sequencing data described in Fig 2. Depicted genes are significantly altered in ZIKV-exposed fetuses of both sexes compared to mock controls, while also differing significantly between sexes within the ZIKV-exposed group. (PDF) [file ppat.1013850.s009.pdf]
