# Supplementary material for: Inapparent maternal ZIKV infection impacts fetal brain development and postnatal behavior
Source: PLoS Pathog. 2026 Jan 12;22(1):e1013850. doi: 10.1371/journal.ppat.1013850 (PMC12822987; doi:10.1371/journal.ppat.1013850)
Supplement: S4 Fig — (PDF) [file ppat.1013850.s004.pdf]

Synaptic-related GO terms	# gene	Fold Change	-Log(padj)
neuron-neuron synaptic transmission	7	7.99	2.13
regulation of synaptic vesicle clustering	6	6.17	1.40
regulation of synapse structural plasticity	6	6.17	1.41
regulation of AMPA glutamate receptor clustering	6	6.17	1.41
presynaptic membrane organization	6	6.17	1.41
translation at postsynapse	26	5.57	7.36
postsynaptic density assembly	7	5.53	1.55
regulation of postsynaptic density assembly	8	5.48	1.82
translation at presynapse	25	5.47	6.93
presynapse assembly	10	5.14	2.26
postsynaptic actin cytoskeleton organization	11	4.92	2.43
synapse maturation	8	4.57	1.49
reg of neurotransmitter receptor at postsynaptic specialization membrane	8	4.33	1.40
positive regulation of long-term synaptic potentiation	12	3.98	2.10
regulation of AMPA receptor activity	10	3.95	1.65
regulation of NMDA receptor activity	10	3.67	1.49
synaptic vesicle maturation	10	3.67	1.49
positive regulation of excitatory postsynaptic potential	14	3.51	2.13
positive regulation of synaptic transmission, glutamatergic	12	2.94	1.32
regulation of postsynaptic membrane neurotransmitter receptor levels	25	2.89	3.09
synaptic transmission, glutamatergic	16	2.84	1.76
regulation of neuronal synaptic plasticity	20	2.78	2.23
synaptic vesicle recycling	18	2.76	1.94
long-term synaptic potentiation	17	2.69	1.53
protein localization to synapse	20	2.67	1.94
positive regulation of synapse assembly	19	2.44	1.56
neurotransmitter secretion	21	2.27	1.46
regulation of neurotransmitter secretion	22	2.2	1.34
