# Supplementary material for: Inapparent maternal ZIKV infection impacts fetal brain development and postnatal behavior
Source: PLoS Pathog. 2026 Jan 12;22(1):e1013850. doi: 10.1371/journal.ppat.1013850 (PMC12822987; doi:10.1371/journal.ppat.1013850)
Supplement: S6 Fig — (PDF) [file ppat.1013850.s006.pdf]

Upregulated DEGs GO terms in NPCs	# gene	Fold Enrichment	-Log(padj)
positive regulation of androgen receptor activity	4	19.38	3.40
snRNA pseudouridine synthesis	3	19.38	2.26
detoxification of hydrogen peroxide	3	19.38	2.26
cellular response to leptomycin B	3	19.38	2.26
premeiotic DNA replication	5	16.15	3.87
electron transport coupled proton transport	4	15.5	2.79
negative regulation of megakaryocyte differentiation	3	14.53	1.75
phenylalanyl-tRNA aminoacylation	3	14.53	1.75
positive regulation of telomerase RNA localization to Cajal body	3	14.53	1.76
positive regulation of establishment of protein localization to telomere	7	13.56	5.11
protein insertion into mitochondrial outer membrane	4	12.92	2.39
positive regulation of cell proliferation involved in heart morphogenesis	4	12.92	2.39
scaRNA localization to Cajal body	3	11.63	1.45
regulation of protein localization to endoplasmic reticulum	3	11.63	1.45
snoRNA guided rRNA pseudouridine synthesis	3	11.63	1.45
mitotic DNA replication initiation	3	11.63	1.45
negative regulation of protein folding	3	11.63	1.46
proton motive force-driven mitochondrial ATP synthesis	37	11.56	28.32
isocitrate metabolic process	4	11.07	2.09
DNA strand elongation involved in DNA replication	7	10.43	4.07
