# Supplementary material for: Inapparent maternal ZIKV infection impacts fetal brain development and postnatal behavior
Source: PLoS Pathog. 2026 Jan 12;22(1):e1013850. doi: 10.1371/journal.ppat.1013850 (PMC12822987; doi:10.1371/journal.ppat.1013850)
Supplement: S7 Fig — (PDF) [file ppat.1013850.s007.pdf]

Downregulated DEGs GO terms in FGNs	# gene	Fold Enrichment	-Log(padj)
mRNA alternative polyadenylation	6	4.82	2.72
phenylalanyl-tRNA aminoacylation	4	4.82	1.57
regulation protein catabolic process at presynapse	4	4.82	1.57
positive regulation of pentose-phosphate shunt	4	4.82	1.57
negative regulation of myofibroblast differentiation	4	4.82	1.57
regulation of termination of RNA polymerase II transcription	4	4.82	1.57
DN4 thymocyte differentiation	4	4.82	1.57
positive regulation of helicase activity	7	4.82	3.32
regulation of microtubule binding	5	4.82	2.13
histone mRNA catabolic process	10	4.38	4.31
nuclear mRNA surveillance	10	4.38	4.31
primary miRNA processing	10	4.38	4.31
CRD-mediated mRNA stabilization	9	4.33	3.73
neg reg of double-strand break repair via nonhomologous end joining	6	4.13	2.07
termination of RNA polymerase II transcription	6	4.13	2.07
TRAMP-dependent tRNA surveillance pathway	6	4.13	2.07
nuclear polyadenylation-dependent rRNA catabolic process	6	4.13	2.07
N-terminal peptidyl-methionine acetylation	6	4.13	2.07
protein import into peroxisome matrix, receptor recycling	6	4.13	2.07
regulation of centriole elongation	6	4.13	2.07
