# Supplementary material for: Inapparent maternal ZIKV infection impacts fetal brain development and postnatal behavior
Source: PLoS Pathog. 2026 Jan 12;22(1):e1013850. doi: 10.1371/journal.ppat.1013850 (PMC12822987; doi:10.1371/journal.ppat.1013850)
Supplement: S1 Table — (PDF) [file ppat.1013850.s010.pdf]

Target	Note	Primer Sequence (5'-3')
Ccl2	Forward	TGGCTCAGCCAGATGCAGT
	Reverse	TTGGGATCATCTTGCTGGTG
Cxcl10	Forward	GCCGTCATTTTCTGCCTCA
	Reverse	CGTCCTTGCGAGAGGGATC
Cxcl1	Forward	ATCCAGAGCTTGAAGGTGTTG
	Reverse	GTCTGTCTTCTTTCTCCGTTACTT
Ifna	Forward	CTTCCACAGGATCACTGTGTACCT
	Reverse	TTCTGCTCTGACCACCTCCC
Tnfa	Forward	AAAATTCGAGTGACAAGCCTGTAGC
	Reverse	GTGGGTGAGGAGCACGTAG
ZIKV	Forward	CCACCAATGTTCTCTTGCAGACATATTG
	Reverse	TTCGGACAGCCGTTGTCCAACACAAG
